# Discerning the Difference Between Lumens and Scalariform Perforation Plates in Impeding Water Flow in Single Xylem Vessels and Vessel Networks in Cotton

**DOI:** 10.3389/fpls.2020.00246

**Published:** 2020-03-10

**Authors:** Yang Gao, Zhenjun Yang, Guangshuai Wang, Jingsheng Sun, Xiaoxian Zhang

**Affiliations:** ^1^Key Laboratory of Crop Water Use and Regulation, Ministry of Agriculture and Rural Affairs, Farmland Irrigation Research Institute, Chinese Academy of Agricultural Sciences, Xinxiang, China; ^2^School of Civil Engineering, University of Wuhan, Wuhan, China; ^3^Department of Sustainable Agricultural Sciences, Rothamsted Research, Harpenden, United Kingdom

**Keywords:** X-ray computed tomography, lattice Boltzmann model, scalariform perforation plates, lumens, hydraulic resistance

## Abstract

The geometrical structure and spatial arrangement of lumens, bordered pits, and scalariform perforation plates in xylem vessels modulate water flow from roots to leaves. Understanding their respective hydraulic functions is essential to unveil how plants regulate their hydraulic networks to facilitate the ascent of sap under biotic and abiotic stresses but is challenging because of the opaque nature of the vessel networks and water flow within them. We made the first-ever effort to discern the difference between lumens and scalariform perforation plates in cotton in impeding water flow in single vessels and vessel networks using X-ray tomography and pore-scale numerical simulation. Three-dimensional structures of xylem vessels in the stem of two cotton cultivars were acquired non-invasively using X-ray computed tomography (CT) at high spatial resolution, and a lattice Boltzmann model was developed to simulate water flow through the xylem networks at micrometer scale. The detailed water velocity and pressure simulated using the model were used to calculate the hydraulic resistance caused by the lumens and the scalariform perforation plates in individual vessels and the vessel networks of the two cotton cultivars. The results showed that the hydraulic resistance spiked whenever water flowed across a perforation plate and that the overall hydraulic resistance caused by the perforation plates in an individual vessel accounted for approximately 54% of the total resistance of the vessel. We also calculated the hydraulic conductance of individual vessels and vessel networks using the simulated water velocity and pressure at micrometer scale and compared it with those estimated from the Hagen Poiseuille (HP) equation as commonly used in the literature by approximating the xylem vessels in the cotton as isolated tubes. While it was found that the HP equation overestimated the hydraulic conductance by more than 200%, the overestimate was largely due to the incapability of the HP equation to represent the perforation plates rather than its approximation of the irregular vessels by circular tubes.

## Introduction

Vascular plants use their xylem networks to ascend water and mineral nutrients from soil to leaves to sustain photosynthesis (Sack and Holbrook, 2006). The xylem vessels contain bordered pits and perforation plates with the former connecting the vessels radially while the latter linking lumens in the axial direction (Lee et al., 2019). The perforation plates and bordered pits are safe valves to mitigate spreading of air bubbles when a plant is under embolization, but they could increase hydraulic resistance to water flow after the embolism is relieved (Choat et al., 2008; Kaack et al., 2019). Understanding the respective resistance caused by lumens and perforation plates is hence essential to unraveling how plants modulate their vessels and vessel networks to best balance the efficiency and safety of their hydraulic networks (Stroock et al., 2014; Gleason et al., 2016) but has been challenging because of the opaque nature of the vessels and water flow within them (Park et al., 2015). Mathematical modeling can play an important part in bridging this gap to help elicit how the hydraulic architectures of plants change under different environments (Mrad et al., 2018; Mencuccini et al., 2019).

The early models for water flow in the xylem network approximated the xylem vessels as hydraulically isolated tubes with water flow in each tube described by the Hagen–Poiseuille (HP) equation (Lewis and Boose, 1995). Although plant physiologists know this is an idealized approximation and overestimates the hydraulic conductance of the xylem network (Sperry et al., 2005; Choat et al., 2008; Melcher et al., 2012), where the errors come from remain obscure. Simplifying the irregular vessels as straight tubes and omitting the perforation plates and bordered pits are known to give rise to errors, but the magnitude of the errors caused by each varied widely from less than 10% (Schulte and Castle, 1993) to more than 200% (Christman and Sperry, 2010). Also, they appear to depend on plant species (Trueba et al., 2019) and the experimental methods used to measure the hydraulic conductance (Chiu and Ewers, 1993).

The past decade has seen an increase in the use of X-ray computed tomography (CT) to non-invasively visualize the embolization of xylem vessels under water stress (Choat et al., 2010, 2016; Cuneo et al., 2016; Nardini et al., 2017; Nolf et al., 2017) as well as the refilling process of the embolism after water stress was relieved (Suuronen et al., 2013; Brodersen et al., 2018). While the X-ray CT has substantially improved our understanding of how the 3D architecture of the xylem vessel network changes in response to abiotic stresses, translating these changes to hydraulic functions remains a challenge because of the difficulty associated with linking the complicated vessel network to water flow within it (Nolf et al., 2017). As a result, in using X-ray images to estimate the hydraulic conductance of xylem vessel network, the vessels were still approximated as isolated tubes with water flow in each described by the HP equation although there has been increased evidence that the hydraulic conductance estimated by the HP equation could be one-order in magnitude larger than that measured from hydraulic-based methods (Nolf et al., 2017).

Water flow in xylem vessels is constrained by their geometrical structures as well as the spatial connectedness of their lumens and perforation plates (Loepfe et al., 2007); it can be directly simulated at scale less than one micrometer if the 3D geometry of the vessel network is known. The past decade has seen a development in pore-scale models to simulate fluid flow in porous materials (Blunt et al., 2013; Zhang et al., 2016) and we have proposed lattice Boltzmann models to simulate water and solute transport in natural and industrial materials at various scales (Zhang and Lv, 2007; Zhang et al., 2010, 2014a,b,c). The purpose of this paper is to combine these pore-scale models with X-ray CT to directly calculate, for the first time, the hydraulic resistances caused by each lumen and perforation plate in single vessels as well as their consequence for hydraulic conductance of the xylem vessel network in cotton. We extracted 3D xylem network in two cotton cultivars by embolizing their xylem vessels first in attempts to contrast their attenuation of X-ray with other tissues, and then directly simulated water flow in their xylem vessels at micrometer scale without making any modification or simplification of the vessel geometries. The water velocity and pressure simulated at micrometer scale was used to calculate the hydraulic resistance caused by each lumen and perforation plate in individual vessels. We also calculated the hydraulic conductance of the vessel networks from the simulated water velocity and pressure and compared it with that estimated by the HP equation as commonly used in the literature by approximating the vessels as isolated straight tubes.

## Materials and Methods

### Acquisition of the Xylem Vessels

We used two cotton cultivars (*Gossypium hirsutum* L.) with one growing in field and one in a phytotron. Although the two cultivars are widely planted in northern China, there is a paucity of research on their xylem vessel anatomy. The cultivar grown in the field was a spring cultivar–Ji863 and its seeds were sown on April 26, 2018. The plants were irrigated with saline water at concentration of 1 g/L on 5 June; 10 days later the plants were sampled predawn by cutting their shoots at 3 cm above the ground surface. The plants were cut using a sharp knife and the stems were immediately wrapped in Parafilm to reduce air invasion. Each stem was cut into 4–5 cm segments under water following the method presented in the literature (Melcher et al., 2012).

The summer cultivar Xinluzhong37 was grown in a phytotron. The seeds were germinated in a seedbed first, and 13 days after the germination the seedlings were transplanted to pots 24 cm high with an internal diameter of 10 cm. The phytotron was controlled at day/night temperature of 28/18°C, relative humidity of 50%, and 12 h of photoperiod from 7.00 am to 7.00 pm with the photosynthetic active radiation of 600 μmol m^–2^ s^–1^ supplied by LED lamps; the lamps were turned off from 7.00 pm to 7.00 am. After transplantation, the pot was irrigated with 1/2 Hoagland nutrient solution for 14 days, and we then added 150 mM of NaCl to the 1/2 Hoagland solution to irrigate the plant once aimed to mimic the soil salinity most cotton in north China is subjected to. After an additional week, we sampled the stem in a similar way as in the field experiment.

The stems of the cottons were scanned using the X-ray CT at the Multiscale Modeling Laboratory of Zhejiang University for X-ray scanning (XT H320, Nikon). Preliminary trials revealed that parameters of 100 kV and 100 μA gave a good-quality image and they were then used in the imaging. The image resolution for Ji863 and Xinluzhong37 was 4.7 μm and 5 μm, respectively. The scanned images were reconstructed using the software provided by the manufacturer and the reconstructed 2D gray slices for each stem were then segmented using the “threshold” method in Image J to extract the xylem vessels. To reduce the noise and increase the contrast, the 2D slices were processed using the Faster Fourier transformation prior to the segmentation. The transverse sections of all vessels were analyzed using the “particle analysis” plug-in in Image J by approximating the cross sections of the vessels as circles. The numbers of these tubes were used to estimate the hydraulic conductance/resistance using the HP equation. To validate that the above methods correctly captured the xylem vessels, we also took scanning electron microscope images of the stems after the scanning with each treatment having three replicas.

### Lattice Boltzmann Simulation

Water flow in the 3D xylem networks extracted from the X-ray images was simulated using the lattice Boltzmann model (LBM) at micrometer scale. The LBM is a mesoscopic method simulating fluid flow by tracking the movement and collision of a number of fictitious particles under constraints that the movement and collisions conserve mass and momentum. The details of the method are given in the Appendix.

**FIGURE 1:**
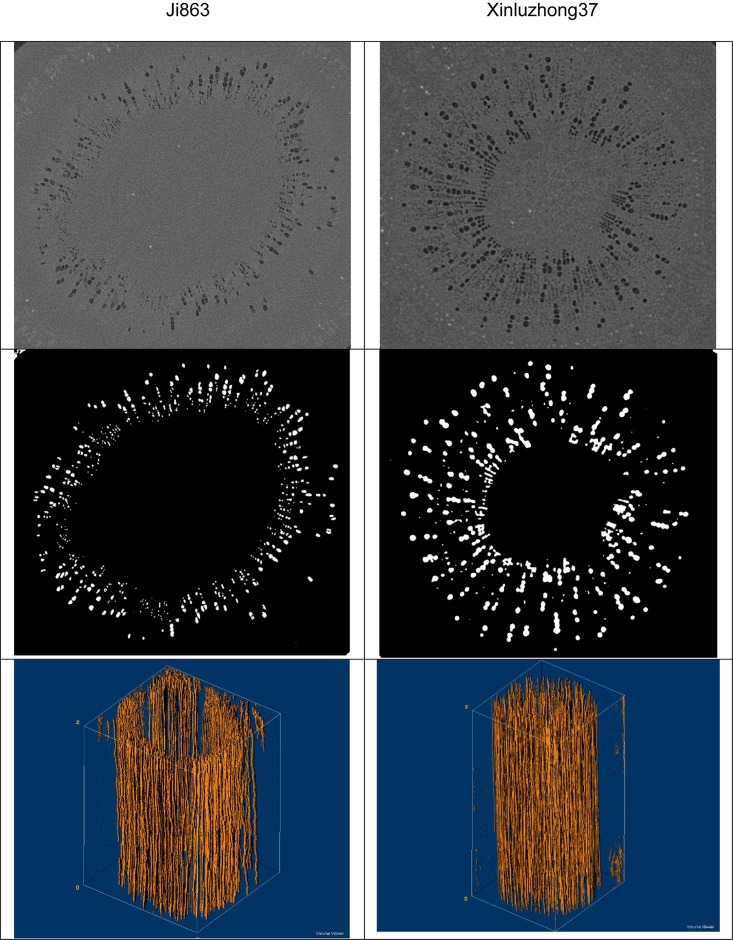
Images of the xylem vessels inside the stems of the two cotton cultivars: **Top row** – transverse sections of the reconstructed gray-scale images. **Middle row** – segmented transverse sections. **Bottom row** – the extracted 3D vessel networks.

Water ascent was driven by a pressure gradient generated by imposing a high pressure on the bottom and a low pressure on the top of each network shown in Figure 1 (Li et al., 2018). Once flow was deemed to have reached steady state, water velocity and pressure at all voxels in the network were sampled and they were then volumetrically averaged across each transverse section to calculate the hydraulic resistance caused by lumens and perforation plates in single vessels and vessel networks, respectively.

The average water flow in the longitudinal direction calculated above was assumed to follow the Darcy’s law (Lewis and Boose, 1995), and the relationship between the average water flow rate and the average pressure is described by:

(1) $$Q\,\left(z\right)=q\,\left(z\right)\,A\,\left(z\right)=-\frac{A\,k}{u}\,\frac{\partial \left(p+\rho \,g\,z\right)}{\partial z}=-K_{a}\,\frac{\partial P}{\partial z}$$

where *Q* is volumetric flow rate (m^3^ s^–1^), *q* is average water velocity (m s^–1^), *A(z)* is area of the transverse section (m^2^), *k* is permeability (m^2^), *u* is dynamic viscosity of the water (m⋅kPa s^–1^), *K*_*a*_ is hydraulic conductance (m^4^kPa^−1^ s^–1^), *p* is pressure (kPa), ρ is water density (kg m^–3^), g is gravitational acceleration (m s^–2^), and z is elevation (m). The hydraulic resistivity is the reciprocal of the hydraulic conductance, i.e., *R = K_*a*_*^–1^, which is the proportionality in the linear relationship between the water pressure gradient and the volumetric flow rate. In all simulations, the water flow rate Q and the average water velocity q were calculated as follows from the water velocity simulated at micrometer scale:

(2) $$\begin{array}{c} Q\,\left(z\right)=\sum_{i=1}^{N_{z}}u_{z}\,\left(x_{i},y_{i},z\right),\\ q\,\left(z\right)=\frac{\sum_{i=1}^{N_{z}}u_{z}\,\left(x_{i},y_{i},z\right)}{A\,\left(z\right)} \end{array}$$

where *N*_*z*_ is the number of the voxels within the transverse section at elevation *z*, *u*_*z*_(*x*_*i*_,*y*_*i*_,*z*) is the vertical water velocity component in the vessel voxel centered on (*x*_*i*_, *y*_*i*_, *z*), *A*(*z*) is the area of the transverse section at elevation *z*. When water flow is in steady state, *Q*(*z*) is a constant and independent of elevation because of the requirement of mass conservation, but *q*(*z*) varies since *A*(*z*) varies in the longitudinal direction.

The transverse section of single vessel varies due to the existence of the perforation plates, and hence its associated hydraulic resistivity also varies in the longitudinal direction. We calculated this variation from the microscopic simulation as follows:

(3) $$R\,\left(z_{i}\right)=-\frac{1}{Q}\,\frac{P\,\left(z_{i}+\delta \right)-P\,\left(z_{i}-\delta \right)}{2\,\delta }.$$

where δ is the side length of the voxels and *P*(*z*) is the average pressure across the transverse section and calculated from:

(4) $$P\,\left(z\right)=\frac{1}{N_{z}}\,\sum_{i=1}^{N_{z}}p\,\left(x_{i},y_{i},z\right),$$

where *p*(*x*_*i*_, *y*_*i*_, *z*) is the water pressure simulated for the vessel voxel centered on (*x*_*i*_, *y*_*i*_, *z*).

The hydraulic conductance of individual vessels and vessel networks can be calculated using the resistance calculated from Eq. (3), but in this work we calculated it directly using the water velocity and pressure simulated at micrometer scale as follows:

(5) $$K_{a}=Q\,\frac{L}{p_{1}-p_{0}},$$

where *p*_1_ and *p*_0_ are the two pressures imposed on the bottom and the top of the network shown in Figure 1, *L* is the height of the network and Q is the volumetric flow rate calculated from Eq. (2).

The above calculations apply to both xylem network and individual vessel. For the network, the variables in the above equations are average over the transverse sections of all vessels, while for individual vessel they are average over the transverse section of the individual vessel only.

## Results

**FIGURE 2:**
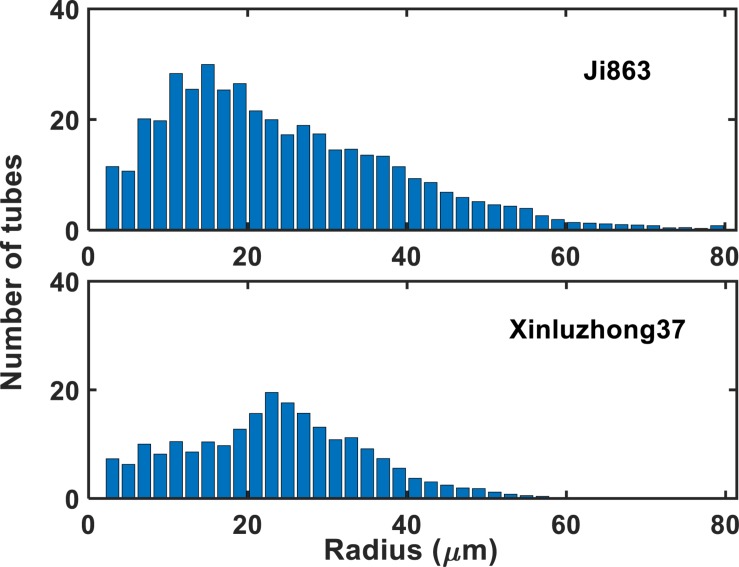
The number of tubes with different radii used to approximate the xylem vessels in the two cotton cultivars.

**FIGURE 3:**
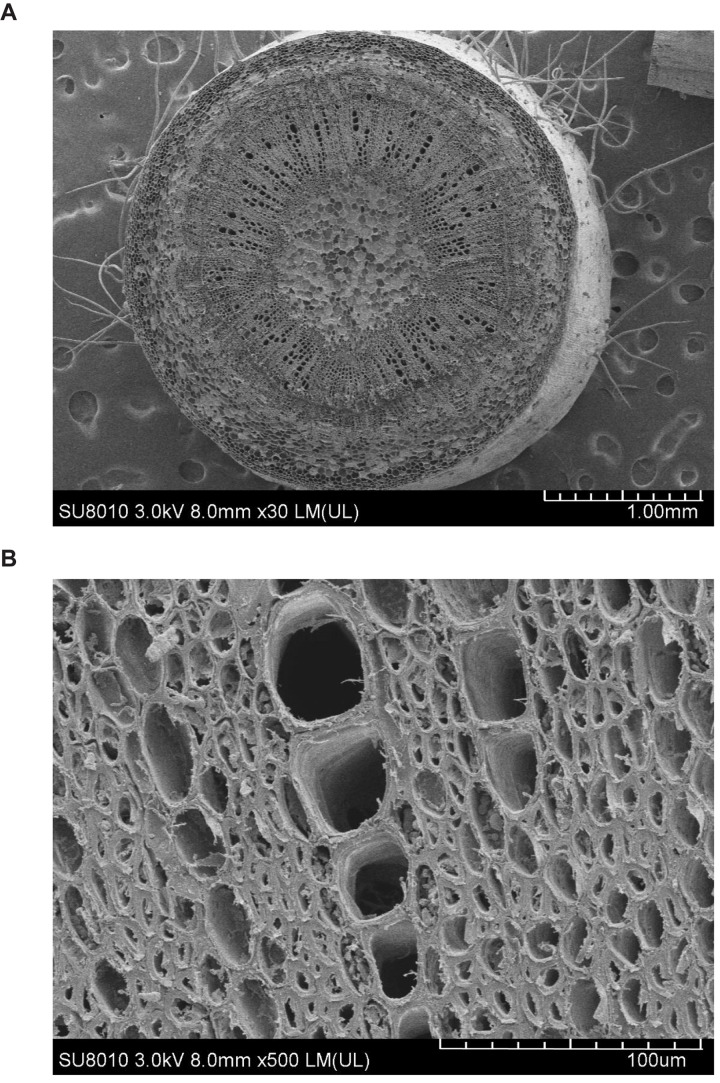
SEMs of the Ji863 cotton cultivar at two contrasting scales show the xylem vessels which are comparable to those captured in the 3D CT image in Figure 1. **(A)** SEM of the stem, **(B)** SEM to highlight the xylem vessels.

Figure 1 shows the contrasting images of the two xylem networks in both 2D and 3D. Figure 2 compares the number of the tubes of different radii used to approximate the vessels in the two networks. As a comparison, Figure 3 shows the SEM of the Ji863 cultivar acquired after the X-ray scanning; the diameter of the xylem vessels exposed in the SEM image was comparable to that estimated from the 3D X-ray image. The lattice Boltzmann method simulated water ascent by applying a high pressure on the bottom and a low pressure on the top of each of the two 3D networks shown in Figure 1.

**FIGURE 4:**
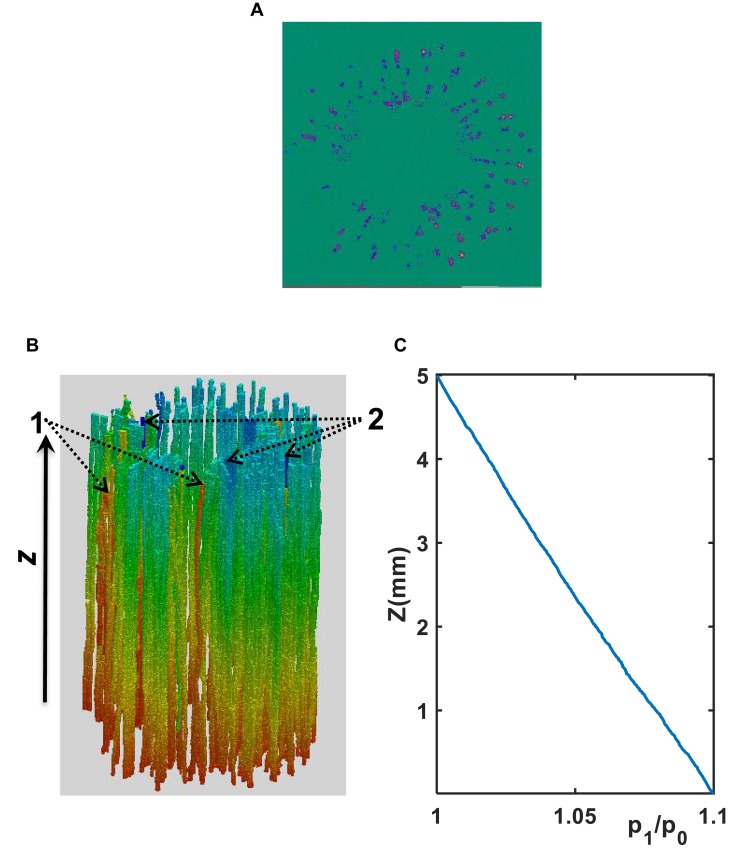
**(A)** A transverse section of water velocity simulated from the LBM (the velocity increases from blue to yellow). **(B)** Pressure distribution (decreases from red to blue) simulated using the LBM at μm scale in the xylem network in the cultivar of Ji863. Vessels pointed by “2” are representative vessels in which the pressure drop is large, while those pointed by “1” are vessels in which the pressure drop is small. **(C)** Distribution of the normalized pressure (p_1_/p_0_) along the longitudinal direction where p_0_ is the constant pressure imposed on the top of the vessel network and p_1_ is the average pressure over the transverse section at location z as shown in Figure 4B.

**FIGURE 5:**
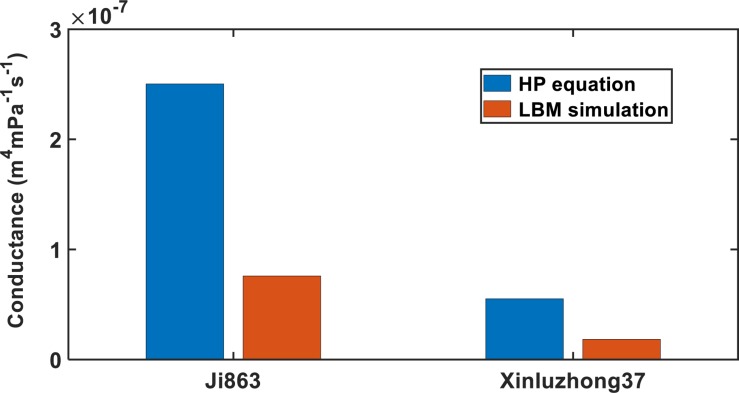
Comparing the Hagen–Poiseuille hydraulic conductance with that calculated directly from the LBM simulation for the xylem networks in the two cotton cultivars.

As an illustrative example, Figure 4 shows the simulated water pressure distribution within the vessel network in Xinluzhong37. Although the applied water pressure on the bottom of all vessels was same, the pressure dissipated differently in different vessels as water ascended because the vessels were interconnected via bordered pits and the transverse section of different vessels varies in different ways in the longitudinal direction. Figure 5 compares the hydraulic conductance of the two vessel networks calculated directly from the simulation with those estimated from the following HP equation by approximating the vessels in each network as isolated tubes:

(6) $$K_{h}\,\left(z\right)=\sum_{i=1}^{N_{z}}\frac{f\,\left(r_{i}\right)\,\pi \,r_{i}^{4}}{8\,u}$$

where *r*_*i*_ is the radius of the tubes, u is the dynamic viscosity of water, *N*_*z*_ is the number of the bins used to group the tubes radii, and *f* (*r*_*i*_) is the number of tubes with radius *r*_*i*_. For both cultivars, the HP equation substantially overestimated the hydraulic conductance, 3.14 times for Xinluzhong37 and 3.39 times for Ji863.

**FIGURE 6:**
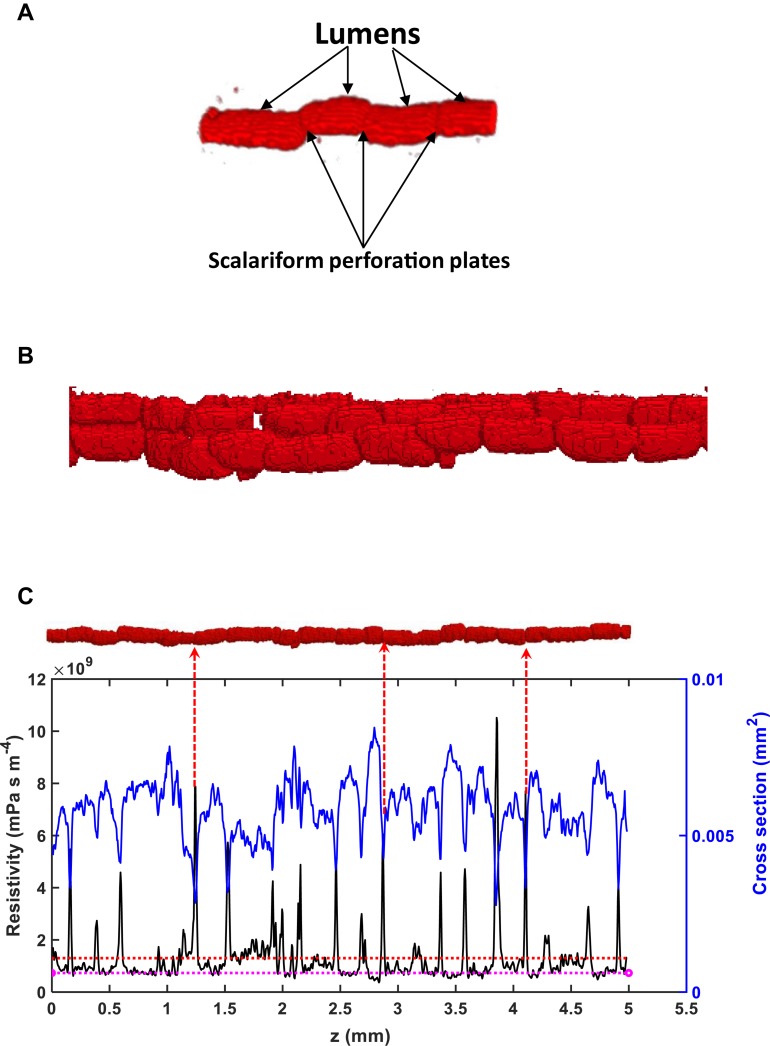
**(A)** A schematic of lumens and scalariform perforation plates in a vessel segment in Ji863 acquired using X-ray CT. **(B)** A schematic of interconnected vessels. **(C)** Change in local resistivity (solid black line), together with change in area of the transverse section of the vessel (solid blue line) on the top of the figure (the red rod), along the longitudinal direction. Also plotted in the figure is the average resistivity of the red rod calculated from the LBM simulation (the red broken line) and the resistivity calculated from the Hagen–Poiseuille equation by approximating the rod as a straight tube with the same volume (the broken pink line). The peaks in resistivity indicate the locations of the scalariform perforation plates, three of which are labeled with red arrows.

The existence of scalariform perforation plates is one cause of the overestimate. To differentiate the hydraulic resistance induced by perforation plates and lumens, we analyzed water flow in individual vessels. As an illustrative example, Figure 6 shows the variation of the hydraulic resistance along a vessel consisting of lumens and scalariform perforation plates; also plotted on the top of Figure 6 is the vessel in which the distance between immediate adjacent perforation plates ranges from 100 μm to 300 μm. It is manifest from the figure that each perforation plat is associated with a spiked resistance, although the amplitude of the spike varies approximately from 2 × 10^9^ s⋅m⋅Pa⋅m^–4^ to 10^10^ s⋅mPa⋅m^–4^. The average resistance caused by all lumens and perforation plates in this vessel was 1.28 × 10^10^ s⋅mPa⋅m^–4^, and is also plotted in the figure for comparison. Figure 6 reveals that the resistances caused by lumens do not show a significant variation and they are all lower than the average resistance, indicating that the perforation plates contributed more to the hydraulic resistance than the lumens. The results for other individual vessels are similar.

**FIGURE 7:**
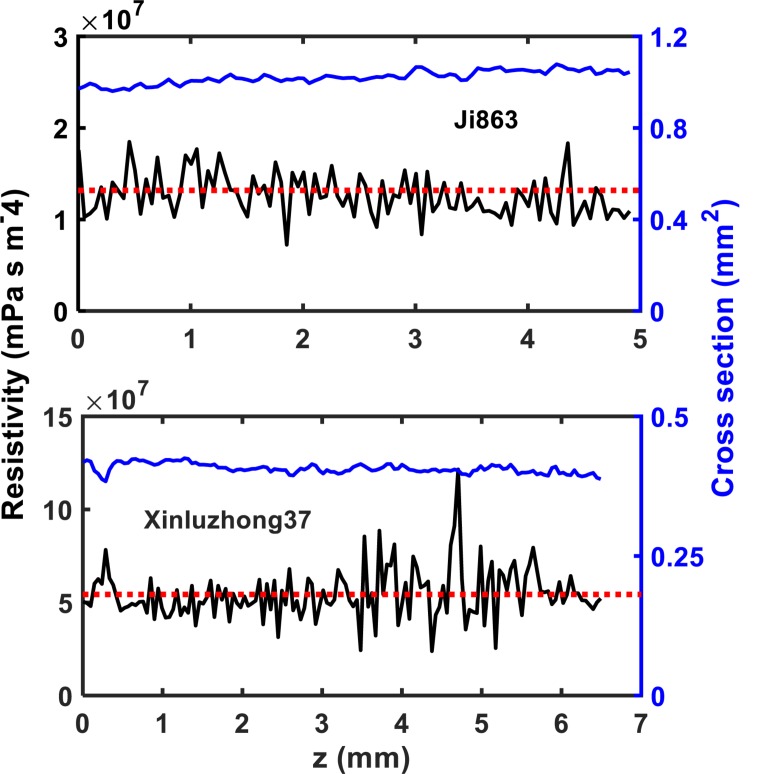
The change in local hydraulic resistivity (the black solid lines) in the xylem network, together with the local transverse sectional area (blue line) of vessel network, along the longitudinal direction for the two cotton cultivars as shown in Figure 2. The red broken line is the average hydraulic resistivity of the xylem network directly calculated from the LBM simulation.

The hydraulic resistance varies erratically in single vessel, spiking at each perforation plate as shown illustratively by the red arrows in Figure 6. Spatial average of the resistances over all vessels in the network can smooth this oscillation. Figure 7 shows the change in average hydraulic resistance of the two networks, along with the transverse sectional area of the networks, in the longitudinal direction for the two cultivars. Although the transverse sectional-area of the vessels in each network is almost constant in the longitudinal direction, its associated hydraulic resistance is not. There is no noticeable relationship between the average hydraulic resistance and transverse sectional area of the vessels for both cultivars.

## Discussion

Modeling water flow in plants from tissues to biosphere has exploded and become instrumental in developing eco-hydrological models since the 1990s. An adequate representation of the hydraulic conductance of xylem vessel networks in all models is essential to their success but is challenging because of the complicated and opaque nature of the xylem vessels. As such, most plant models idealized the xylem vessels as isolated straight tubes to calculate their hydraulic conductance using the HP equation. Although it was commonly understood in plant physiology community that such approximations give rise to errors because their incapability of distinguishing the lumens and perforation plates, the magnitude of the errors remained obscure. The perforation plates vary widely from species to species, but the lumens in most vascular plants are geometrically similar. How the types of perforation plates affect the accuracy of HP model for estimating hydraulic conductance of the xylem vessel network is poorly understood. This paper is to bridge this knowledge gap by taking cotton as an example and showing how to use X-ray CT and pore-scale modeling to study water flow in single vessels and vessel networks in the stem of two cotton cultivars.

### Single Vessels

The X-ray images can differentiate the scalariform perforation plates and the lumens but are unable identify the bordered pits because of the limitation of the spatial resolution (4–5 μm). Despite this, our simulation results provide some insights into how the lumens and the perforation plates combine to modulate water flow in single vessels.

It was found that every perforation plate spikes the hydraulic resistance although the magnitude of the spikes varies between the plates (Figure 6). As most vessels are interconnected and it is difficult to isolate all them. For those that can be isolated, visual inspection of the change in simulated hydraulic resistance and the X-ray image revealed that the number of the perforation plates was approximately 30/10 mm for Xinluzhong37 cultivar. For Ji863 cultivar, the resolution of the X-ray images was not high enough to identify all the perforation plates.

Hydraulic-based experimental apparatuses are unable to distinguish the resistances caused by each perforation plate, and they instead separate the perforation plates from the lumens by approximating the vessel as a straight tube and calculating the hydraulic resistance of the perforation plates as the difference between the resistance estimated from the HP equation for the tube and that measured from the hydraulic-based method (Christman and Sperry, 2010). To evaluate the consistence of our results with experimental data in the literature, we assume that the combined length and average resistivity of the lumens and the perforate plates in a single vessel are *L* and *R*, respectively. If the average resistivity and length of all lumens in the vessel are *L*_*r*_ and *R*_*L*_, respectively, and the average resistivity of all perforation plates is *R*_*p*_, we have the following relationship:

(7) $$R=L_{L}\,R_{L}\,/\,L+\left(L-L_{L}\right)\,R_{P}\,/\,L.$$

For the vessel shown on top of Figure 6, its average resistivity is *R* = 1.28 × 10^9^ mPa s m^–4^. Using the cross-sectional area of 0.05 mm^2^ as a threshold to separate the lumens and the perforation plates, the lengths of all perforation plates sum to be 0.84 mm and the lengths of all lumens sum to be 4.16 mm; we can use these to separate the resistance caused by all lumens from that induced by all perforation plates. From Figure 6, the average resistivity of the lumens is 7.01 × 10^8^ mPa s m^–4^ and we therefore have *R* = 0.832*R*_*L*_+0.168R_*P*_. Thus, the combined contribution of all perforation plates to the vessel resistivity is 1.28–0.832*R*_*L*_ = 6.93 × 10^8^ mPa s m^–4^, which accounts for 54% of the vessel resistivity. This is consistent with the results reported in the literature for species including ginkgo, bracken, willow and pepper tree (Sperry et al., 2005), indicating that approximating the lumen as a tube might not give rise to an unacceptable error if the role of the perforation plates is correctly represented. However, there was also evidence that approximating the single xylem vessels in branch of white ash tree (*Fraxinus americana*) accurately predicted their longitudinal conductance (Zwieniecki et al., 2001), probably due to the lack or weak impact of scalariform perforation plates in this species.

If we approximate the vessel on top of Figure 6 by a tube, its radius is 43 μm and the associated hydraulic resistivity is 7.34 mPa s m^–4^–close to the average resistivity of the lumens (also plotted in Figure 6), implying that the overestimate of the HP equation is due to its incapability of representing the scalariform perforation plates rather than approximating the irregular vessel as a straight tube (Chiu and Ewers, 1993; Hong et al., 2017).

**FIGURE 8:**
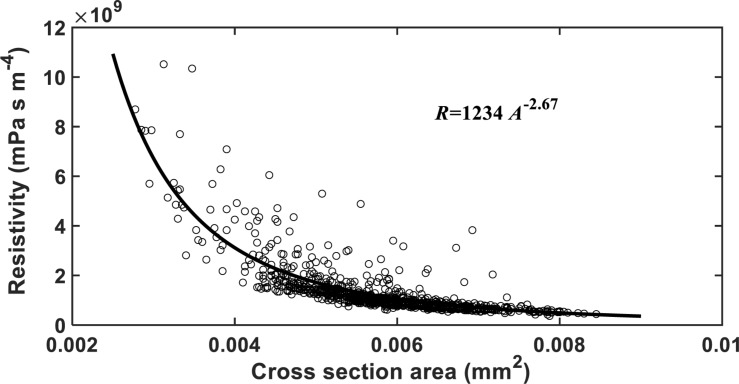
The change in hydraulic resistivity with the transverse sectional area of single vessels in the studied cotton cultivars.

The HP equation predicts a vessel resistivity that is proportional to the cross-sectional area of the vessel up to the second power. This scaling law does not apply to the vessels with scalariform perforation plates as studied in this paper. The resistivity of the vessels calculated from the LBM simulation is proportional to its transverse sectional area (including lumens and perforation plates) up to the −2.26 power (Figure 8). This is anticipated as the transverse section of the vessel is not circular, and its resistance is hence greater than predicted by assuming it to be circular. In addition to this, the non-linear pressure distribution in the proximity of the perforation plates due to a sudden change in cross sectional area is another reason underlying the deviation from the second power.

### Vessel Networks

**FIGURE 9:**
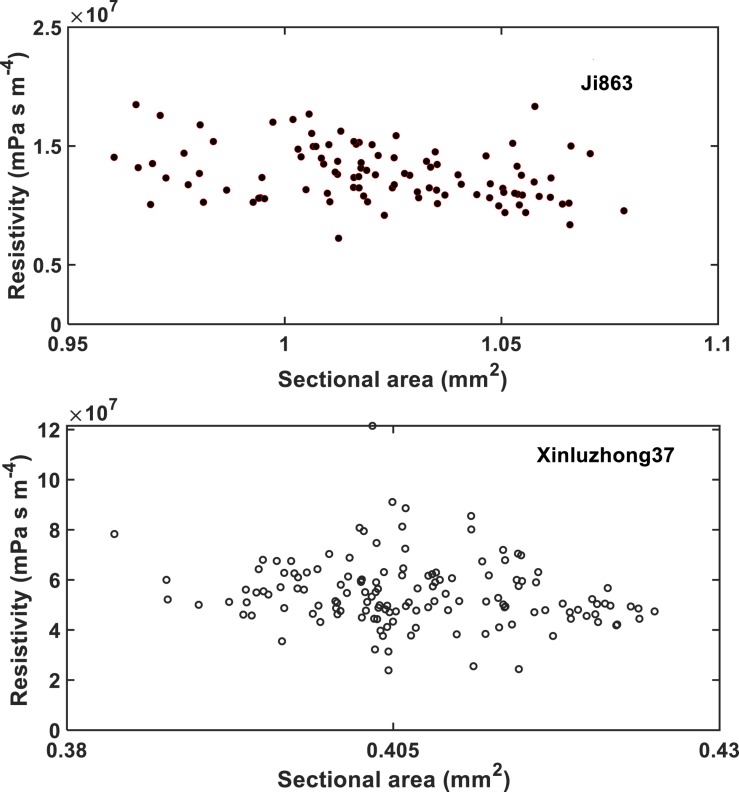
The change in hydraulic resistivity with the transverse sectional area of the xylem network in the two cotton cultivars.

The simulation results at micrometer scale for single vessels are consistent with the existing experimental results in the literature, but the relationship between the hydraulic resistance and anatomical features of a single vessel cannot be scaled up to the vessel networks for the two cotton cultivars studied in this paper, due to that the shapes of the lumens and the perforation plates vary from vessel to vessel and that some vessels were interconnected (Figure 4). The transverse sectional areas of the vessels in the stem of the two cultivars differ slightly from each other (Figure 1). There is no correlation between the cross-sectional area and its associated resistivity for neither network (Figure 9) because some vessels in the network are likely interconnected (Figures 1, 6), making water flow in the network more complicated than in a single vessel. It is manifest from Figure 4 that although the applied pressure on the bottom of the network was the same for all vessels, the pressure dissipated differently between the vessels as water ascended. Figure 4 shows that the water pressure dropped more quickly in some vessels (marked by 1) than in others (marked by 2) and that the pressure distribution in single vessels could be non-linear. However, the volumetric average over the transverse section of the network smooths these vessel-vessel variations and yields an average pressure that changes approximately linearly along the longitudinal direction (Figure 4C). Therefore, the average water ascent over the transverse section of the xylem network can still be described by the Darcy’s law in which the average resistance is proportional to the average volumetric flow rate.

The inaccuracy of the HP equation is largely due to its inability to represent the perforation plates rather than its approximation of the irregular vessels. As the perforation plates vary from species to species (Trueba et al., 2019), the errors of the HP equation also vary. For the two cotton cultivars the relative error of the HP equation was 235% for Ji863 and 210% for Xinluzhong37, larger than the errors reported in Christman and Sperry (2010) for 13 species with different scalariform perforation plates.

### Comparison With Literature Results Measured From Hydraulic-Based Method

The hydraulic conductance estimated from the HP equation for the single vessel in Figure 6 is 1.36 × 10^–9^ m4 mPa^–1^ s^–1^, which is 1.74 times the hydraulic conductance (2.25 × 10^–10^ m4 mPa^–1^ s^–1^) calculated directly from the simulation. For the xylem network, however, this ratio increases to 3.14 for Xinluzhong37 and 3.39 for Ji863. This appears to be consistent with existing experimental results for grass (Martre et al., 2000, 2001) and Salvia mellifera (Hargrave et al., 1994), both showing that the hydraulic conductance measured using the hydraulic-based methods was approximately one third of that estimated by the HP equation. However, there is no consensus about if there is a congruence between the hydraulic conductance estimated by the HP equation and the real hydraulic conductance. Recent study using X-ray tomography indeed showed that the vulnerability curves estimated by the HP equation using the vessel radii estimated from X-ray images agreed reasonably well with those measured from hydraulic-based methods (Nolf et al., 2017). This is possible only when the hydraulic conductance estimated and measured by the two methods are linearly correlated and independent of the vessel diameters. However, there is also contradictory evidence that the hydraulic conductance estimated from the HP equation was non-linearly related to that measured from hydraulic methods (Martre et al., 2001). As a result, the vulnerability curve estimated using the X-ray CT and the HP equation differed markedly from that measured from the hydraulic-based method (Savi et al., 2017) in that the hydraulic conductance estimated by the former could be more than 20 times that measured by the latter (Trueba et al., 2019). Given that the hydraulic-based experimental methods are prone to errors (Cochard et al., 2013) and need great care in preparing the samples (Choat et al., 2010), it remains obscure whether these inconsistent results are generic or due to artificial errors. Microscopic simulations might have the edge as it can correctly reproduce water flow at micrometer scale in each vessel if the 3D vessel network is adequate. This has become feasible with the rapid development in X-ray CT and its application in plant physiology over the past few years (Brodersen and Roddy, 2016; Scoffoni et al., 2017; Wason et al., 2017). For example, a X-ray micro-CT is capable of acquiring images at spatial resolution of less than 1.0 μm (Koddenberg and Militz, 2018). We anticipate that with the increase in the use of X-ray tomography, numerical models capable of simulating fluid flow at micrometer scale will play an important role not only in calculating hydraulic resistance but also in unraveling how the scalariform perforation plates combine with the bordered pits to work as safe valves to modulate embolism spreading when plant is under stress as well as embolism refilling after the stress is relieved (Brodersen et al., 2018; Lee et al., 2019). This is readily achievable using the two-phase lattice Boltzmann model which has been advanced substantially over the past decade (Chen et al., 2014).

Separating the xylem vessels from other tissues using X-ray CT needs to contrast their attenuations of the X-ray, which is not trivial when the vessels are filled with water. Embolizing the vessels is a method to contrast their attenuation of the X-ray, but embolization could shrink the vessels. Also, the xylem vessels are geometrically complicated, and it is difficult to know *a priori* if a designed embolization can embolise all vessels. All these could impact the results in using the proposed methods.

## Conclusion

X-ray CT and the lattice Boltzmann model were used to simulate water flow at micrometer scale in the xylem network in the stem of two cotton cultivars. The xylem vessels were embolized prior to scanning using X-ray CT in attempts to contrast their attenuation of X-ray with other tissues. Water ascent in the network was simulated using the lattice Boltzmann model under a pressure gradient, and the simulated water velocity and pressure at micrometer scale were used to analyse the resistance induced by perforation plates and lumens in single vessels and vessel networks, respectively. For single vessels, the resistance spikes at each perforation plate although the magnitude of the spikes varies between the plates. Overall, the perforation plates account for 54% of the vessel resistance and the lumens contribute 46%. For the xylem networks, its average hydraulic conductance calculated from the microscopic simulation is approximately one third of that estimated from the HP equation. The spatial resolution of the X-ray images is not high enough to differentiate the bordered pits, but their impact on water flow appears to have been captured, manifest by the roughness of the vessel wall in the X-ray CT images. Our results shed some light on how the geometrical structure of single xylem vessels affects water flow as well as the consequence for water ascent in the vessel network.

Simulating water flow in 3D vessel networks acquired using X-ray CT image does not make assumptions to the vessel geometry and is hence most accurate. However, it needs high-resolution images to capture the detailed vessel anatomy. This limits the size of the sample that the method can deal with.

## Data Availability Statement

The datasets generated for this study are available on request to the corresponding author.

## Author Contributions

YG and JS designed the experiment. YG and GW conducted the field and lab experiment. ZY was responsible for X-ray tomography imaging. XZ analyzed the X-ray images, ran all simulations, and wrote the draft manuscript.

## Conflict of Interest

The authors declare that the research was conducted in the absence of any commercial or financial relationships that could be construed as a potential conflict of interest.

## Appendix

Water flow in the xylem network was simulated using the lattice Boltzmann (LB) method at micrometer scale by tracking the movement and collision of a number of fictitious fluid particles under constraint that their movement and collision conserve mass and momentum. The propagation of each particle in vessels is described by the following multiple-relaxation time lattice Boltzmann equation (d’Humieres et al., 2002):

$$f_{i}\,\left(\text{x}+\delta \,t\,{\text{e}}_{i},t+\delta \,t\right)=f_{i}\,\left(\text{x},t\right)+M^{-1}\,S\,M$$

(A1) $$\left[f_{i}^{e\,q}\,\left(\text{x},t\right)-f_{i}\,\left(\text{x},t\right)\right],$$

where *f*_*i*_(x, *t*) is the particle distribution function at location ***x*** and time *t* moving at lattice velocity ***e****_*i*_*, δ*x* is the size of the voxels in the image, δ*t* is time step, $f_{i}^{e\,q}\,\left(\text{x},t\right)$ is equilibrium distribution function, *M* is a transform matrix and *S* is the collision matrix. The product M*f* in Eq. (1) transforms the particle distribution functions to a moment space in which the collision operation$m=S\,M\,\left[f_{i}^{e\,q}\,\left(\text{x},t\right)-f_{i}\,\left(\text{x},t\right)\right]$ is performed. The post-collision result in the moment space is then transformed back to particle distribution functions by *M*^−1^*m*. We use the D3Q19 lattice model in this paper where the particle distribution functions move in 19 directions with 19 velocities: (0, 0, 0), (±δ*x*/δ*t*, ±δ*x*/δ*t*, 0), (0, ±δ*x*/δ*t*, ±δ*x*/δ*t*), (±δ*x*/δ*t*, 0, ±δ*x*/δ*t*) and (±δ*x*/δ*t*, ±δ*x*/δ*t*, ±δ*x*/δ*t*) (Qian et al., 1992). The collision matrix is diagonal and the terms in it are given as follows:

$$S=(s_{0},s_{1},s_{2},s_{3},s_{4},s_{5},s_{6},s_{7},s_{8},s_{9},s_{10},s_{11},$$

$$s_{12},s_{13},s_{14},s_{15},s_{16},s_{17},s_{18}){}^{T},$$

$$s_{0}=s_{3}=s_{5}=s_{7}=0,$$

$$s_{1}=s_{2}=s_{9-15}=1/\tau ,$$

(A2) $$s_{4}=s_{6}=s_{8}=s_{16-18}=8\,\left(2-\tau^{-1}\right)/\left(8-\tau^{-1}\right)$$

The fluid simulated by the above model has a kinematic viscosity μ = δ*x*^2^(τ−0.5)/6δ*t* and pressure *p* = ρδ*x*^2^/3δ*t*^2^.

The equilibrium distribution functions in Equation A1 are defined as follows:

$$f_{i}^{e\,q}=w_{i}\,\left[\rho +\rho_{0}\,\left(\frac{3\,{\text{e}}_{i}\cdot \text{u}}{c^{2}}+\frac{9\,{\left({\text{e}}_{i}\cdot \text{u}\right)}^{2}}{2\,c^{4}}-\frac{3\,\text{u}\cdot \text{u}}{2\,c^{2}}\right)\right]$$

$$w_{0}=1/3,$$

$$w_{i}=1/18,||{\text{e}}_{\text{i}}||=\delta \,x/\delta \,t$$

(A3) $$w_{i}=1/36\;||{\text{e}}_{\text{i}}||=\sqrt{2}\,\delta \,x/\delta \,t\,$$

where *c* = δ*x*/δ*t* and ρ_0_ is a reference fluid density to ensure that the fluid is incompressible when the flow is in steady state (Zou et al., 1995). The bulk fluid density ρ and velocity *u* are updated after each time step by:

$$\rho =\sum_{i=0}^{18}f_{i},$$

(A4) $$\rho_{0}\,\text{u}=\sum_{i=1}^{18}f_{i}\,{\text{e}}_{i}.$$

Implementation of the above model consists of two steps to advance a time step. The first one is to calculate the collision in the moment space and then transform the results back to particle distribution functions, i.e., to calculate $f_{i}^{*}=f_{i}\,\left(\text{x},t\right)+M^{-1}\,S\,M\,\left[f_{i}^{e\,q}\,\left(\text{x},t\right)-f_{i}\,\left(\text{x},t\right)\right]$; and the second step is to move the post-collision particular distribution function $f_{i}^{*}$ to position at x + δ*t*e_*i*_ during the time period of δ*t*. During the streaming step, whenever $f_{i}^{*}$ hits a voxel that is not air-filled, it is bounced back to where it was before the streaming to represent the hydrophilic xylem wall where the bulk sap velocity is zero.

Fluid flow in the air-filled network was driven by a pressure gradient in the longitudinal direction created by imposing two different constant pressures: one on the top and one on the bottom of the image. Once flow was deemed to have reached steady state, we sampled water velocity and pressure in all voxels in the image and they were used to calculate hydraulic conductance and resistance of the xylem network and individual vessels.
